# Refinement of Strut-and-Tie Model for Reinforced Concrete Deep Beams

**DOI:** 10.1371/journal.pone.0130734

**Published:** 2015-06-25

**Authors:** Mohammad Panjehpour, Hwa Kian Chai, Yen Lei Voo

**Affiliations:** 1 Department of Civil Engineering, Faculty of Science, Engineering, Technology and Mathematics (FOSTEM), INTI International University, 71800, Nilai, Malaysia; 2 Department of Civil Engineering, Faculty of Engineering, University of Malaya, 50603, Kuala Lumpur, Malaysia; 3 DURA Technology Sdn Bhd, Ipoh, Malaysia; University of Zaragoza, SPAIN

## Abstract

Deep beams are commonly used in tall buildings, offshore structures, and foundations. According to many codes and standards, strut-and-tie model (STM) is recommended as a rational approach for deep beam analyses. This research focuses on the STM recommended by ACI 318-11 and AASHTO LRFD and uses experimental results to modify the strut effectiveness factor in STM for reinforced concrete (RC) deep beams. This study aims to refine STM through the strut effectiveness factor and increase result accuracy. Six RC deep beams with different shear span to effective-depth ratios (a/d) of 0.75, 1.00, 1.25, 1.50, 1.75, and 2.00 were experimentally tested under a four-point bending set-up. The ultimate shear strength of deep beams obtained from non-linear finite element modeling and STM recommended by ACI 318-11 as well as AASHTO LRFD (2012) were compared with the experimental results. An empirical equation was proposed to modify the principal tensile strain value in the bottle-shaped strut of deep beams. The equation of the strut effectiveness factor from AASHTTO LRFD was then modified through the aforementioned empirical equation. An investigation on the failure mode and crack propagation in RC deep beams subjected to load was also conducted.

## Introduction

Deep beams are mainly used in tall buildings, offshore structures, and foundations [1]. According to ACI 318–11, a deep beam has a clear span equal to or less than four times the overall depth. Regions with concentrated loads within twice the member depth from the face of support are also considered deep beams [2].

Based on many codes and standards, the strut-and-tie model (STM) is recommended as a rational approach for analyzing and designing reinforced concrete deep beams [2–8]. The concepts of the STM are originally referred to truss analogy proposed by Ritter [9] and Mörsch [10] as well as compression field theory proposed by Michael P.Collins [11] for the design of shear structural elements. The truss model was then brushed up by Schlaich et al and proposed for consistent design of reinforced concrete structures [12]. Since 2002, ACI building code has been adopted the STM and replaced it with previously rather simple equation for design and analysis of deep beams [2]. STM is based on the lower-bound theorem which states that distribution of stresses used to resist an applied load is safe, as long as equilibrium is satisfied through structural elements and all parts of structure have stress less than yield [13]. Prior research have been attempted to evaluate the plastic behaviors of compressive strut in STM in cracked concrete fields [14].

**Fig 1:**
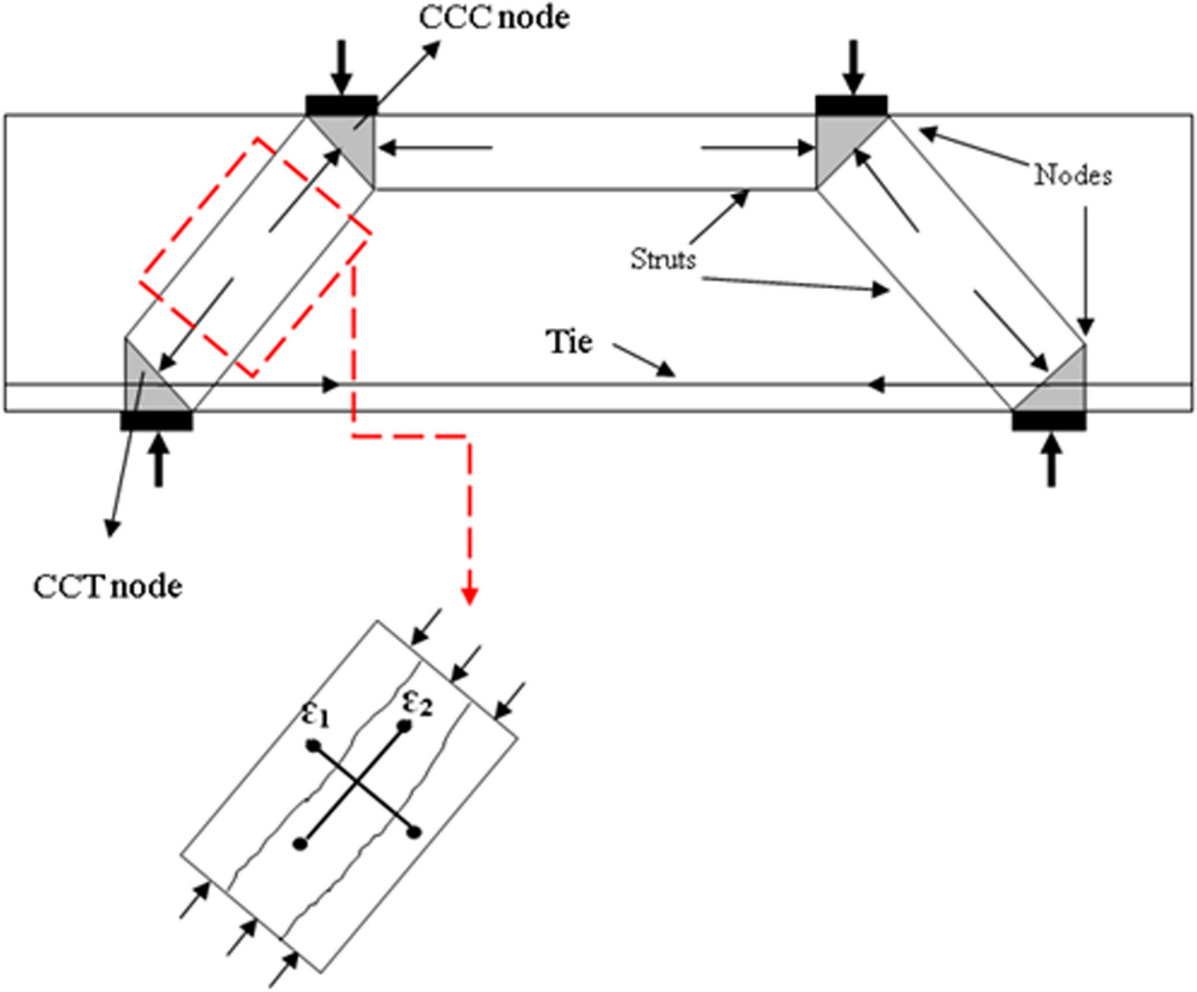
STM for deep beam with principal strains in inclined concrete strut.

Numerous codes and standards have proposed equations to predict concrete strut effectiveness factor by considering various variables that affect discontinuity region (D-region) behaviour. In the present study, these codes and standards are classified into two groups regarding the proposed STM method [15, 16]. The first group comprises AASHTO LRFD, CSA-S6-06, and AS3600, which define the strut effectiveness factor as a function of the tensile strain of tie and angle between strut and tie [3, 4, 17]. The foregoing effectiveness factor is originally derived from research on modified compression-field (MCF) theory, which proposed the stress-strain relationship for cracked concrete in compression [11]. Fig 1 illustrates the STM for deep beams, which elaborates the principal strains in strut.

The second group comprises ACI 318–11, DIN 1045–1, and NZS 3101, which recommend a constant value for the strut effectiveness factor depending on the grad of concrete and the satisfaction of the required reinforcements [2, 6, 8].

AASHTO LRFD recommends Eqs (1) and (2) to determine the strut effectiveness factor.

$$\varepsilon_{1}=\varepsilon_{\text{s}}+(\varepsilon_{\text{s}}+0.002){\text{cot}}^{2}(\alpha )\;\left(\text{AASHTO LRFD eq}.\;5.6.3.3.3-2\right)$$ (1)

$${\text{f}}_{\text{ce}}=\frac{{\text{f}\prime }_{\text{c}}}{0.8+170\varepsilon_{1}}<0.85{\text{f}\prime }_{\text{c}}\;\left(\text{AASHTO LRFD eq}.\;5.6.3.3.3-1\right)$$ (2)

Where,

ɛ_1_: principal tensile strain of strut, ɛ_s_: tensile strain in an adjoining tie, α:angle between adjoining tie and strut in strut-and-tie model (*rad*), f_ce_: effective compressive strength of strut in STM along the strut centreline (*MPa*), f’_c_: specified concrete compressive strength (*MPa*),

The effectiveness factor equation derived from Eq (2) is shown below.

$$\lambda =\frac{1}{0.8+170\varepsilon_{1}}<0.85$$ (3)

Where,

λ: strut effectiveness factor

Numerous studies have been conducted on the behavior of reinforced concrete (RC) deep beams with openings [18], RC deep beams subjected to combined bending and axial forces [19], and RC deep beams using different grade of concrete [19–22]. Other researchers have also explored the effects of support settlement on deep beam and STM behavior [23]. The latest research conducted on RC deep beam has investigated and compared the cracking moment and rupture modulus in deep beams [24]. The effect of carbon fiber-reinforced polymer (CFRP) strengthening on energy absorption of deep beam has been currently investigated [25]. Application of STM for CFRP strengthened deep beams has been also researched [26, 27].

The present study examines the STM that ACI 318–11 and AASHTO LRFD recommended and uses experimental results to modify the strut effectiveness factor for RC deep beams. Non-linear finite element modelling (FEM) is applied in the present study because codes and standards [2, 3] recommend both STM and non-linear FEM for deep beams analysis. Unlike other studies conducted on STM [28–34], the present research proposes the strut effectiveness factor using the experimental values of the principal tensile strain in RC strut. Thus, the present study aims to establish an empirical relationship between the values of principal tensile strain perpendicular to the strut centerline from the experiment and those proposed by AASHTO LRFD Equations. The experimental program also investigates the width and propagation of cracks in RC deep beams as well as the corresponding failure mode.

## Methodology

This study was supported by High Impact Research Grant UM.C/625/1/HIR/MOHE/ENG/54. Six RC deep beams with shear span to the effective-depth (a/d) ratios of 0.75, 1.00, 1.25, 1.50, 1.75, and 2.00 were constructed in this experiment. The strain in mid-length of the inclined strut perpendicular to the strut centerline was measured during the test. According to the prior research, addition of transverse reinforcement beyond the required amount of reinforcement does not increase the ultimate shear strength of deep beams. Using extra transverse reinforcement only provides a marginal strength for deep beams [1, 35]. In this research, the required amount of transverse reinforcement was provided based on ACI 318–11 and AASHTO LRFD (2012).

### 2.1 Details of deep beams

**Fig 2:**
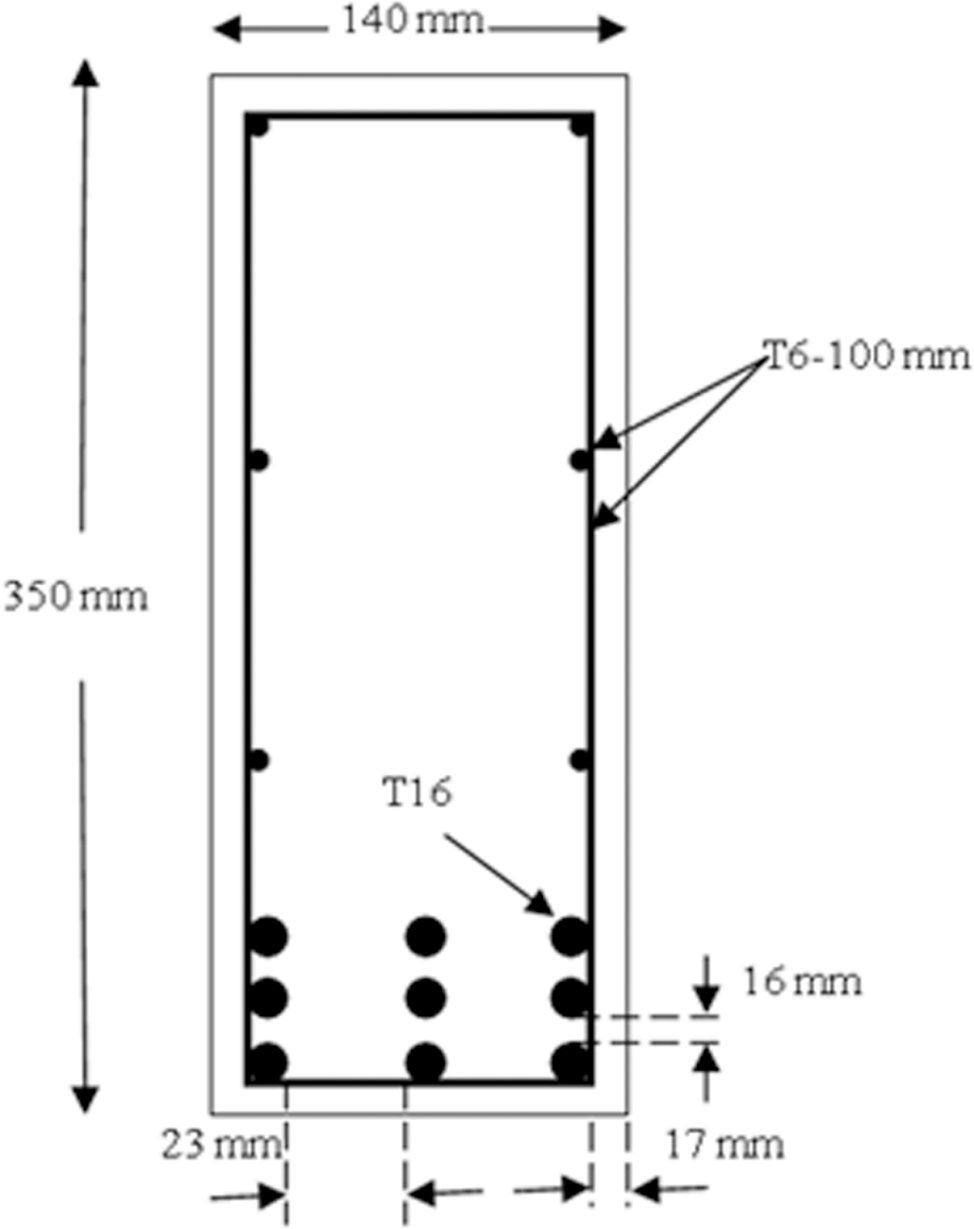
Beams cross section details.

**Fig 3:**
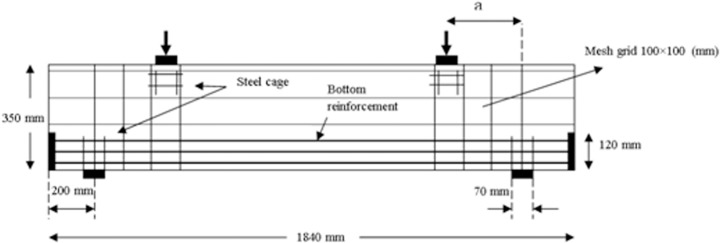
Typical reinforcement details.

The deep beams were identical in every aspect except for the position of the steel cages. Each beam had a length of 1840 mm with a rectangular cross-section as indicated in Fig 2. The flexural reinforcement consisted of 9T16 deformed steel bars, which were placed in three layers at the bottom of the beam cross-section. These steel bars were welded into 10 mm thick steel plates at both beam ends to provide adequate anchorage capacity. The anchorage steel plates that had a height of 120 mm fully covered the beam widths. An orthogonal steel mesh reinforcement with diameter of 6 mm and spacing of 100 mm was provided as the transverse reinforcement. This reinforcement provided the required minimum amount of web reinforcement that ACI 318–11 and AASHTO LRFD recommend [2, 3]. The additional reinforcements (steel cages) were provided under the load plates and on top of the support plates to prevent premature local bearing stress, as illustrated in Fig 3. The presence of steel cages and anchorage plates may change the formation of compression-compression-tension (CCT) node on top of the support plates. However, the formation of CCT node is not the concern of this research and does not affect strut performance. The beams were cast using a single supply of ready-mixed concrete. The maximum aggregate size in concrete was 10 mm. Two types of coarse (uncrushed granite) and fine (uncrushed sand) aggregates were used in the mix design. The water-to-cement ratio was 0.47. The concrete slump was 60 mm. The mix design for one cubic meter of concrete is shown in Table 1.

**Table 1:** Concrete mix design.

	Cement	Water	Fine agg.	Coarse agg.	Super plasticizer (1/m^3^)
**Quantities(kg)**	340	160	855	1045	20

The support and load plates fully covered the area beneath and on top of the beam with 70 mm width and 10 mm thickness. The deep beams were tested 28 days after casting. The cylinder compressive strength and cylinder splitting tensile strength of concrete were 37.02 MPa and 3.31 MPa from the test, respectively. The yield strength of reinforced steel bar was 440 MPa from the test according to ASTM-E8.

### 2.2 Test procedure and instrument

**Fig 4:**
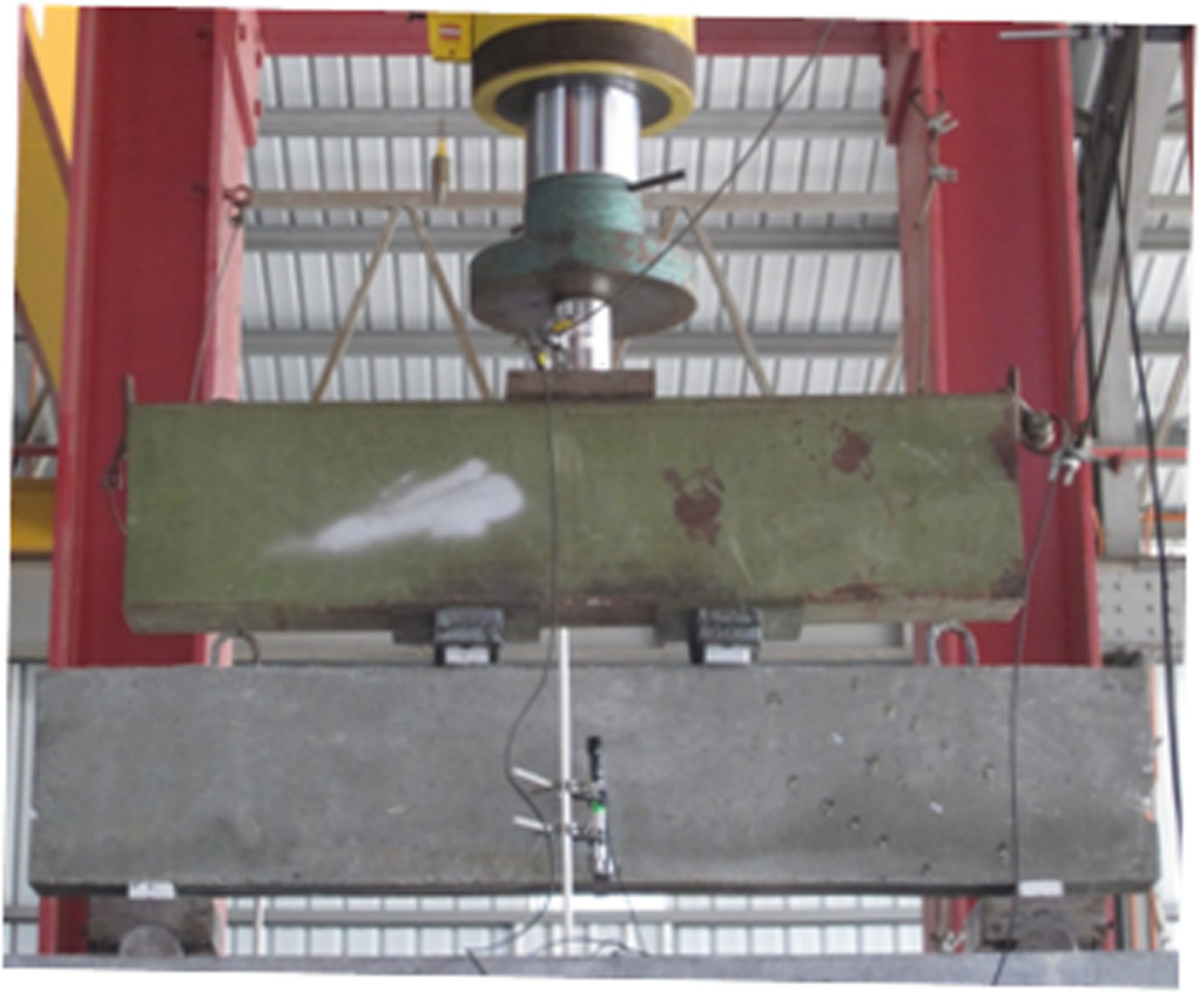
Test set-up.

**Fig 5:**
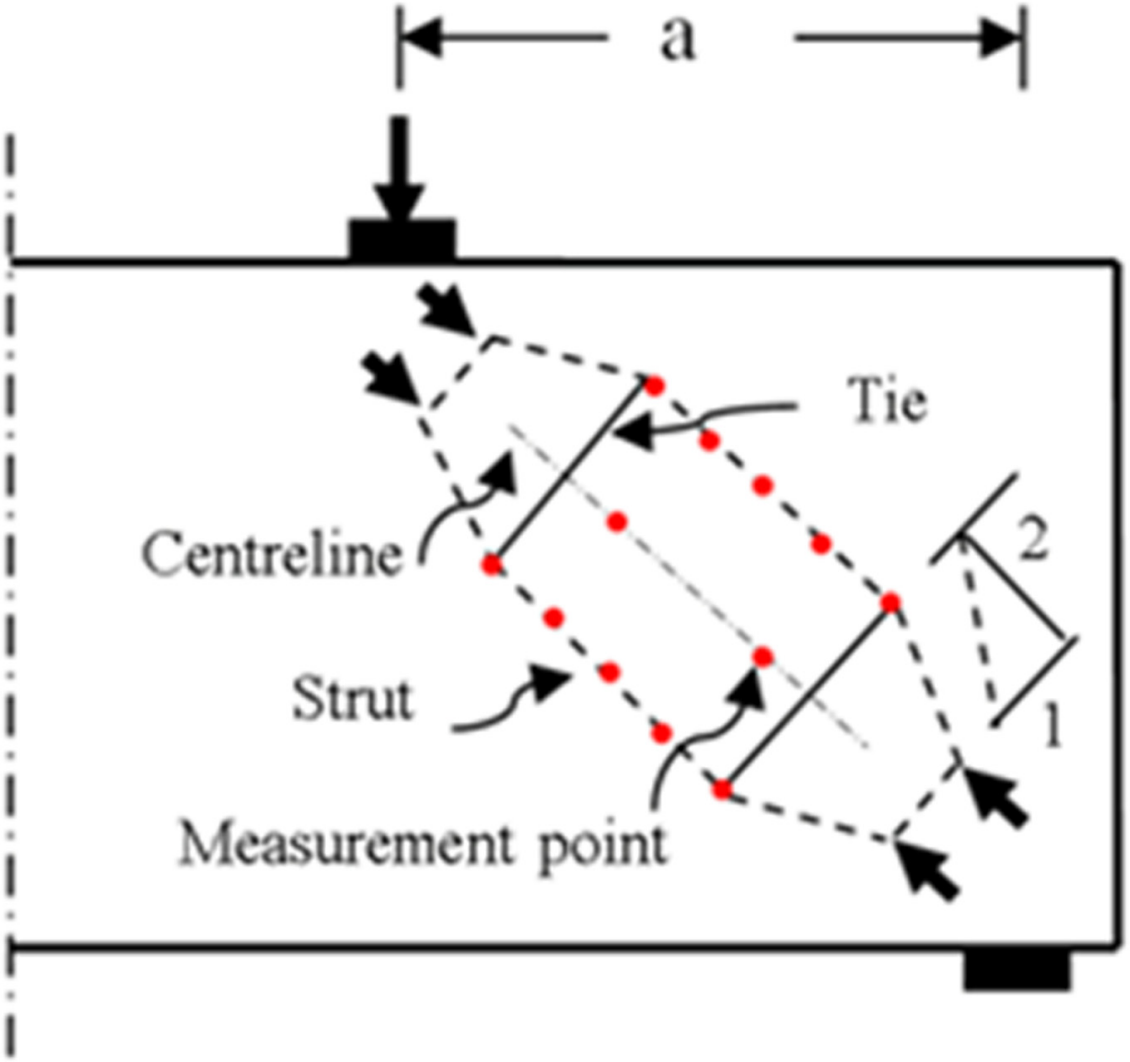
Strain measurement points on strut.

The beams were tested until they failed under a four-point load set-up, as shown in Fig 4. The load was uniformly applied with a controlled loading rate until failure using a hydraulic actuator with a maximum capacity of 5000 kN. Fig 5 illustrates the position of strain measurement points on the strut with a 200-mm distance equal to the demountable mechanical strain gauge (DEMEC) bar. The DEMEC discs were installed on the strain measurement points along and perpendicular to the strut centerline. The resolution of DEMEC gauge was 0.001 mm. DEMEC measured the strain along and perpendicular to the strut centerline to perceive the bottle-shaped strut deformation. However, the strain value perpendicular to the strut centerline at mid-height of the beam cross-section was the main concern of this research. A portable microscope with resolution of 0.02 mm was utilized to measure the cracks width in the D-region of deep beams surfaces.

## Experimental Results and Discussion

**Fig 6:**
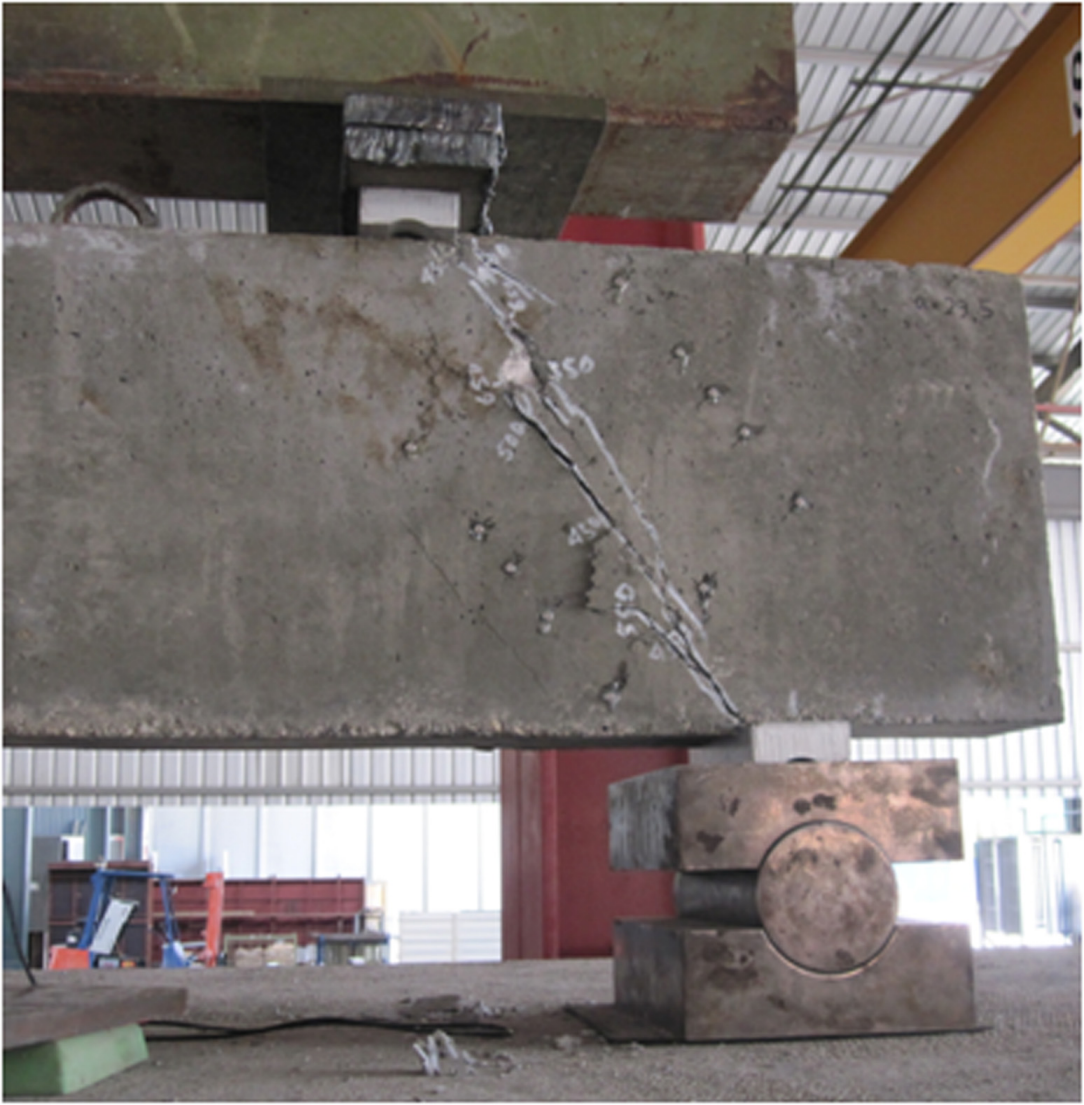
Typical crack pattern of RC deep beam.

The strut represents concrete compressive stress fields with the compression stresses acting parallel to the strut centreline. Thus, the cracks formation parallel to the strut centerline, as indicated in Fig 6, implies the strut formation while loading. The diagonal crack that propagated between the load and support plates, as shown in Fig 6, is due to the principal tensile stress perpendicular to the strut centerline.

### 3.1 The failure modes observed for deep beam

Few concepts are considered as the basis of RC deep beam shear failure mode. According to these concepts, once inclined cracking starts to propagate, the RC deep beam with shear reinforcement behaves as a truss with concrete between two alternative inclined cracks and the shear reinforcement acting as strut and tie, respectively [1]. The concrete crushing mode in the compressive zone was not observed in this experiment because the compressive zones under the loading plates and on top of the support plates were reinforced using steel cages. In accordance with the beam design, the adequate longitudinal steel bars were assembled to prevent steel bars from yielding. During loading, diagonal cracks were observed to be propagating towards the loading and support plates. Shear failure mode was dominant for all the tested deep beams. This result was expected based on the prior research [1]. Fig 6 illustrates the typical shear failure mode and crack pattern observed in RC deep beams in the experiment.

### 3.2 Crack width of RC deep beams

According to the experimental observations, the elastic behavior dominated in the RC deep beams by approximately 30% of the ultimate load. Then the diagonal cracks firstly appeared in the mid-depth of the beams and subsequently extended towards the load and support plates. A further load increase widened and extended the existing diagonal cracks, whereas new diagonal cracks appeared. The flexural cracks appeared at the imposed load, which was greater than approximately 50% of the ultimate load. The flexural cracks hardly reached mid-depth of the beams sections. Finally, one of the diagonal cracks perceptibly extended and widened at approximately 90% of the ultimate load. Consequently, final failure of the beams occurred.

**Fig 7:**
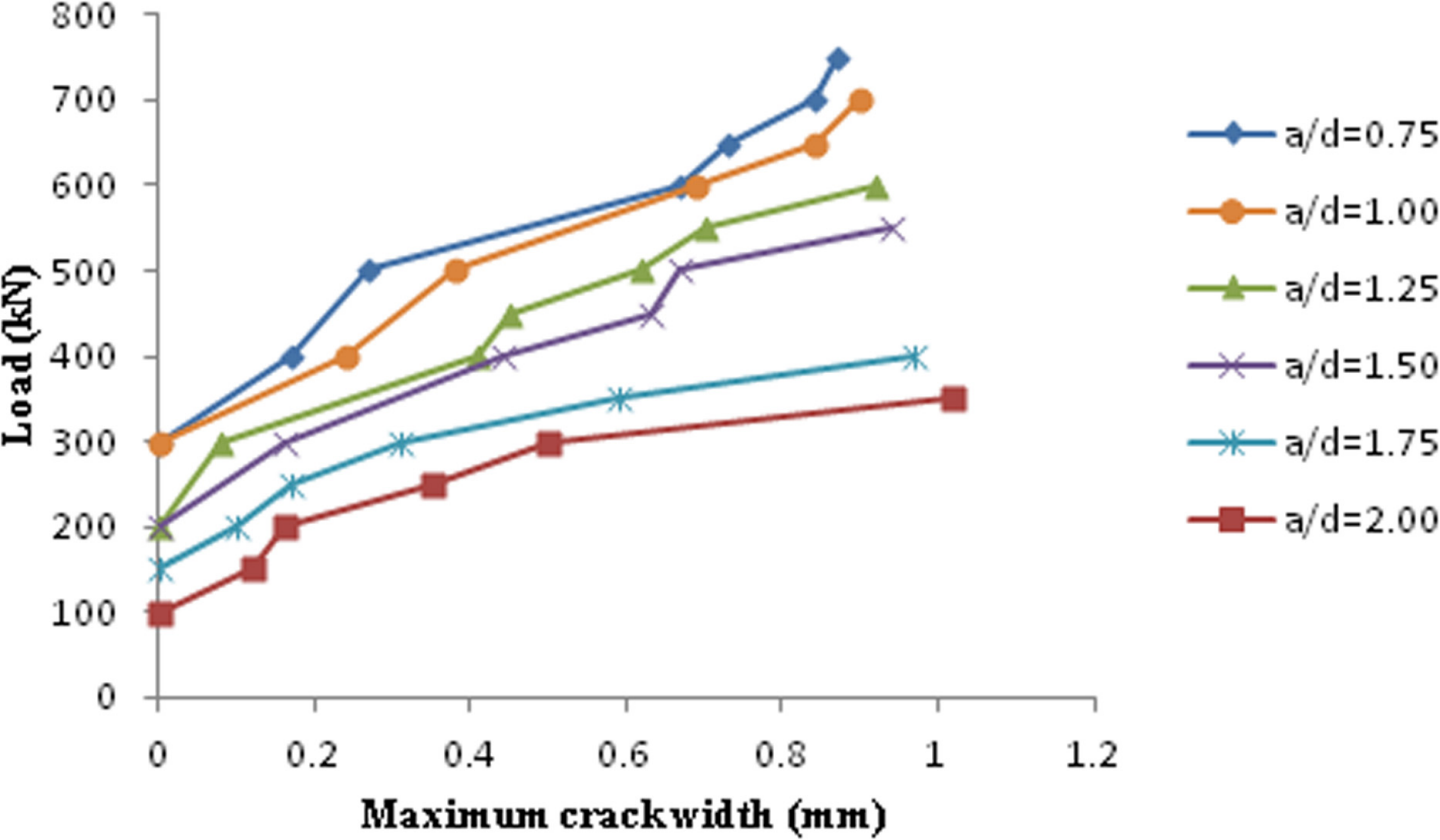
Maximum width of diagonal cracks in RC deep beams at different applied loads and a/d.

The maximum diagonal crack width for RC deep beams and the corresponding loads were measured up to approximately 95% of the ultimate loads, as illustrated in Fig 7. In addition, as the a/d ratio increased the maximum width of diagonal crack, which was measured in each step of the load increment, increased. For an equivalent imposed load, the maximum width of the diagonal crack increased with the increase of the a/d ratio.

### 3.3 STM and non-linear FE modeling of deep beams

According to ACI 318–11 and AASHTO LRFD, both STM and non-linear finite element modeling (NLFEM) are recommended for deep beams analysis. Thus, the ultimate shear strength of deep beams obtained from NLFEM and STM recommended by ACI 318–11 as well as AASHTO LRFD (2012) are compared with experimental results in the following sections.

#### 3.3.1 Non-linear finite element modeling

ABAQUS software was used for non-linear FE analysis using three-dimensional (3D) modeling of the RC deep beams. The beams were modeled using solid geometry for concrete, sweep geometry for steel bars, and shell geometry for CFRP sheets with extrusion option. The C3D20R element with 20 nodes was used for the mesh configuration of 3D modeling. Perfect bond between concrete and steel bars was assumed in the modeling. A mesh size of 5 mm that resulted from the convergence test was used in the NLFEM for the mesh size dependency. Due to the symmetry of the geometry, loading, boundary conditions and material properties, a quarter of deep beams were assembled in NLFEM. The use of a quarter model significantly reduced the computational time. A static uniformly distributed load was applied on the load plates.

This study adopted suitable configurations to define the non-linear behaviours of steel and concrete in NLFEM. The softening behaviour of the concrete in compression attributed to transverse cracking was an important mechanism that affected NLFEM results. Therefore, Eqs (4) and (5) were used in NLFEM for the ascending and descending branches of the modified Hagenstd’s stress-strain curve. The modified Hagenstd’s stress-strain includes asoftening coefficient ζ which Belarbi and Hsu proposed [36, 37].

$$\sigma =\varsigma {\text{f}}_{\text{c}}^{\prime }\left[\frac{2\varepsilon }{\varepsilon_{0}}-{\left(\frac{\varepsilon }{\varepsilon_{0}}\right)}^{2}\right]\;\varepsilon \leq \varepsilon_{0}$$ (4)

$$\sigma =\varsigma {\text{f}}_{\text{c}}^{\prime }\left[1-0.15{\left(\frac{\varepsilon -\varepsilon_{0}}{\varepsilon_{\text{u}}-\varepsilon_{0}}\right)}^{2}\right]\;\varepsilon_{0}\leq \varepsilon \leq \varepsilon_{\text{u}}$$ (5)

$$\text{Where}\;\varsigma =\frac{0.9}{\sqrt{1+400\varepsilon_{1}}}$$ (6)

Where,

σ: concrete compressive stress in Hognestad’s model (*MPa*), δ: modified coefficient, 0.92 for concrete with compressive strength greater than 35, ɛ: concrete compressive strain in Hognestad’s model, ɛ_0_:peak compressive strain of concrete, ɛ_u_: ultimate compressive strain of concrete, ɛ_1_: principal tensile strain of strut

**Fig 8:**
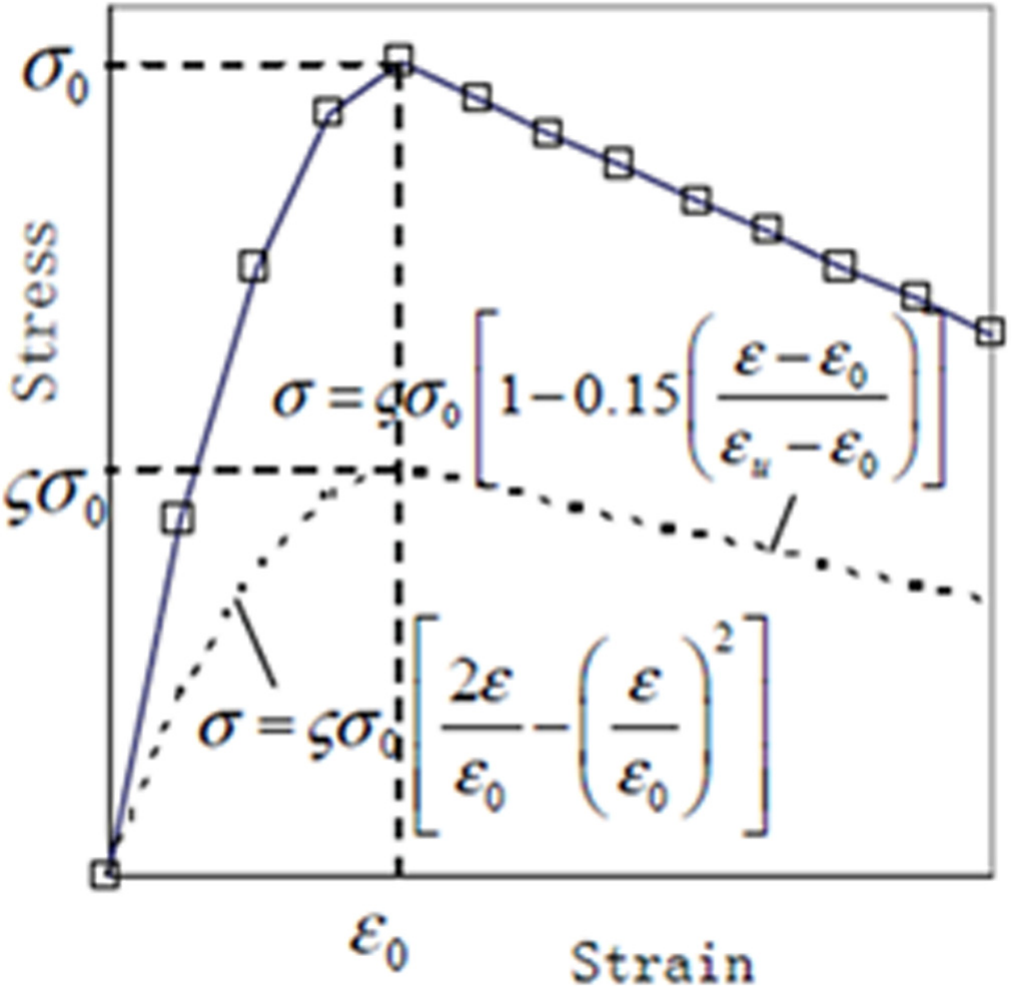
Plotted modified Hognestad stress-strain curve of concrete in this study.

The parameters below that resulted from the test were used for the modified Hognestad stress-strain curve of concrete as shown in Fig 8.

ɛ_0_: 0.002, ɛ_u_ = 0.003, σ_0_ = 37.02 (*MPa*), δ = 0.92

Where, σ_0_: peak concrete compressive stress in Hognestad’s model (*MPa*)

The parameters below that resulted from the test were used to define the input data of the concrete properties in the ABAQUS software.

w_c_ = 2420 (*kg/m* ^*3*^), E_c_ *=* 31146.53 (*MPa*), f_t, split_ = 3.31 (*MPa)*, f_r_ = 4.21 (*MPa*), υ_c_ = 0.2

where, w_c_: density of concrete (*kg/m* ^*3*^), E_c_ = elastic modulus of concrete (*MPa*),f_t,split_: concrete tensile strength from split test (*MPa*), f_r_: concrete modulus of rupture (*MPa*), ν_c_: concrete Poisson’s ratio

The input data for steel properties that resulted from the test comprising elastic modulus, yield stress, and Poisson’s ratio are as follows.

Es = 200,000 (*MPa)*, f_y_ = 440 (*MPa)*, ν_s_ = 0.3

Where, E_s_: elastic modulus of steel (*MPa)*, *f*_*y*_: *yield strength of steel*, *ν*_*s*_ *= steel Poisson’s ratio*

**Fig 9:**
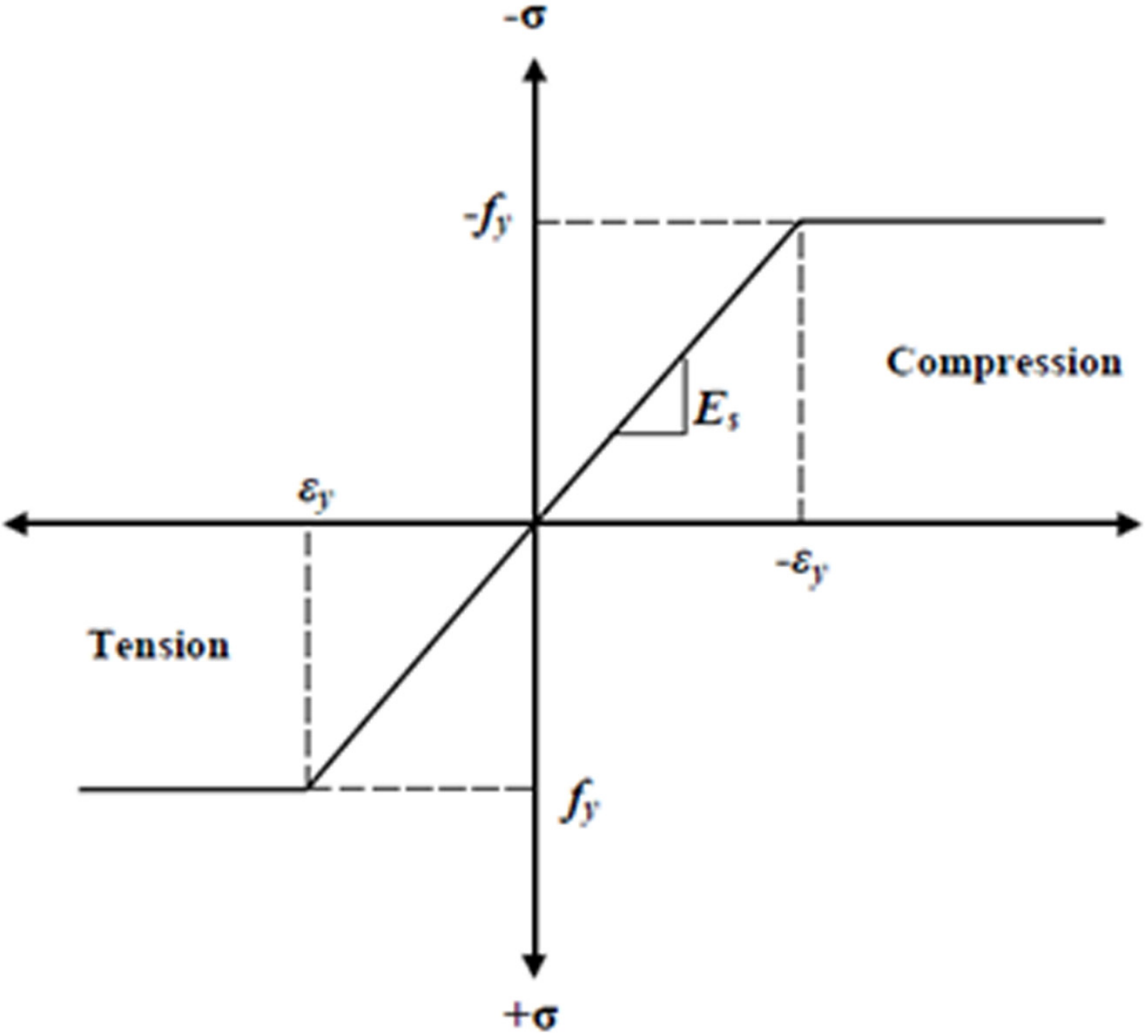
Stress-strain curve for steel reinforcement.

The steel for the finite element models was assumed to be an elastic-perfectly plastic material and identical in tension and compression. Fig 9 shows the stress-strain relationship for steel reinforcement used in this study.

**Fig 10:**
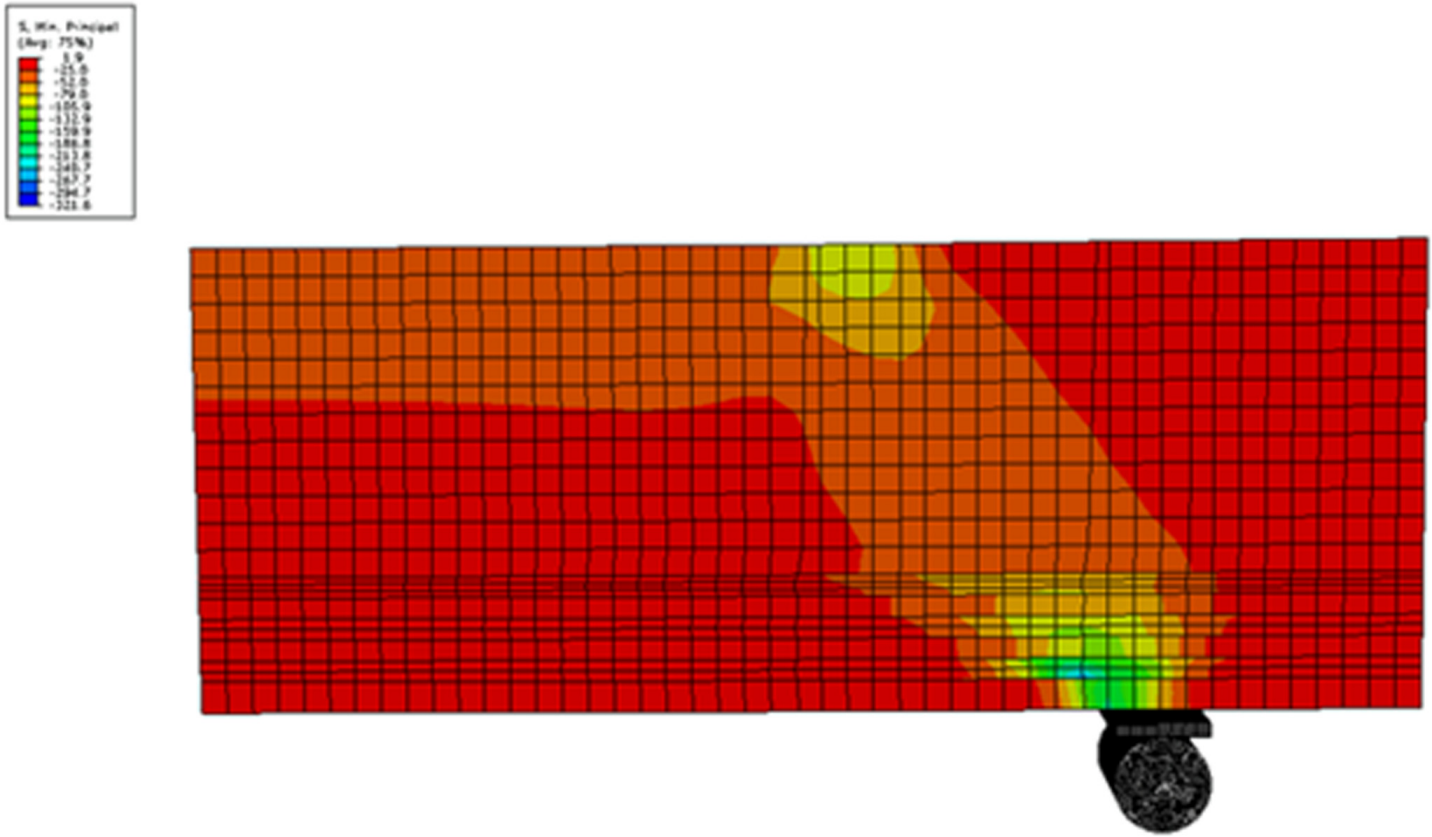
Typical FE compressive stress-distribution in RC deep beams.

A few assumptions were made in NLFEM, as highlighted below. The Poisson’s ratio was constant throughout the loading history. The concrete behavior was linear prior to cracking and non-linear after cracking. The steel was an elastic-plastic material and identical in tension and compression. Decisions were carefully made regarding mesh layout use, representation of reinforcement details, elements type, loading method, support conditions, convergence criteria, and adoption of material behavior models to attain a convergence of results. The typical compressive stress-distribution that resulted from NLFEM for RC deep beams is illustrated in Fig 10.

The mesh size in this figure was considered 20 mm to prevent disordering of figure. However, the mesh size of 5 mm resulted from the convergence test was used in NFEM in this study.

#### 3.3.2 STM method proposed by ACI 318–11 and AASHTO LRFD

The STM illustrated in Fig 1 was used to analyze RC deep beams. The effective strength of concrete in RC strut was calculated using Eq (7).

$${\text{f}}_{\text{ce}}=0.85\beta_{\text{s}}{\text{f}}_{\text{c}}^{\prime }\;\left(\text{ACI Eq}.\;\text{A}-3\right)$$ (7)

According to ACI 318–11, Section A.3.3, for structural elements with reinforcement satisfying

$$\beta_{\text{s}}=0.75$$

Thus, the value of the strut effectiveness factor is

$$\lambda ={\text{f}}_{\text{ce}}/{\text{f}}_{\text{c}}^{\prime }=0.85\times 0.75=0.6375$$

**Fig 11:**
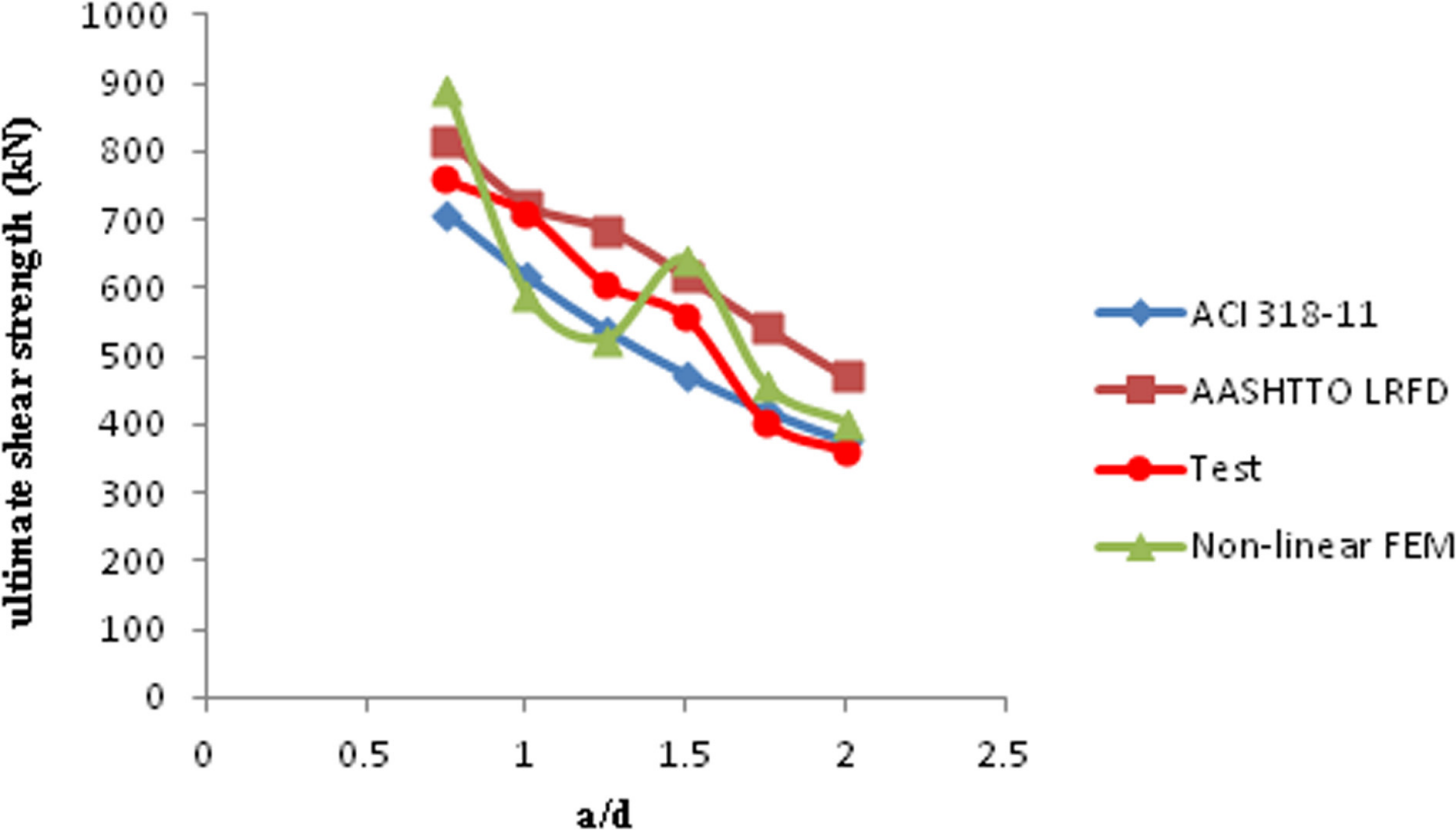
Comparison of shear strength of deep beams from codes and experiment.

Eq (5) was used to calculate the strut effectiveness factor for the AASHTO LRFD STM. The ultimate shear strength of deep beams that resulted from the ACI 318–11, AASHTO LRFD, NLFEM, and the experiment were compared, as shown in Table 2 and Fig 11(P_ACI_: ultimate shear strength of deep beam from STM recommended by ACI 318–11 (*kN*), P_AASHTO_: ultimate shear strength of deep beam from STM recommended by AASHTO LRFD (*kN*), P_FEM_: ultimate shear strength of deep beam from non-linear finite element modeling (*kN*), P_test_: ultimate shear strength of deep beam from the experiment (*kN*)).

**Table 2:** Comparison of ultimate shear strength of RC deep beams resulted from ACI 318–11, AASHTO LRFD, NLFEM and experiment.

a/d	a(mm)	P_ACI_ (kN)	P_AASHTO_ (kN)	P_FEM_ (kN)	P_test_(kN)
0.75	216.75	707.95	817.18	892.34	756.95
1.00	289.00	615.77	720.51	588.11	709.02
1.25	361.25	537.38	685.97	525.49	604.08
1.50	433.50	472.88	617.00	641.49	555.91
1.75	505.75	420.17	541.14	455.91	403.01
2.00	578.00	376.88	470.01	401.32	360.00

Based on Table 2, the results of the AASHTO LRFD STM in the range of 0.75 ≤ a / d ≤ 1.5 were averagely in better agreement with the experimental results than the results of ACI 318–11. However, the results of the ACI 318–11 STM were in better agreement with the experimental results than the results of AASHTO LRFD in the range 1.75 ≤ a / d ≤ 2. Thus, the AASHTO LRFD STM may provide more accurate results for deep beams (0.75 ≤ a / d ≤ 1.5) than ACI 318–11. Non-linear FEM was also used to predict the ultimate shear strength of RC deep beams. However, the results of non-linear FEM showed lower accuracy than the results obtained through the recommended STMs by ACI 318–11 and AASHTO LRFD. This condition is attributed to the assumptions made in non-linear FEM.

The AASHTO LRFD STM was selected for modification because this STM obtained better results for very deep beams than ACI 318–11 and non-linear FEM. The principal tensile strain value of strut mainly governs the strut effectiveness factor equation of AASHTO LRFD. Accordingly, the next section aims to modify the AASHTO LRFD STM through the principal tensile strain value of strut.

### 3.4 Refinement of STM recommended by AASHTO LRFD (2012)

Eqs (1) and (2) were originally derived from the research conducted on modified compression-field theory [11]. The principal tensile strain of strut (ɛ_1_) used in Eqs (1) and (2) includes the crack widths because MCF theory was proposed, particularly for cracked RC. The amounts of principal tensile strain at the mid-length of strut (ɛ_1-test_), which are given in Table 3, were measured in the experiment using a DEMEC gauge with a resolution of 0.001 mm. P_ɛ1-test_:ultimate shear strength of deep beam calculated by STM using ɛ_1-test_ (*kN*).

**Table 3:** Principal tensile strain of strut resulted from test and AASHTO LRFD method.

a/d	ɛ_s_	ɛ_1-AASHTO_	ɛ_1-test_	ɛ_1-test_ / ɛ_1-AASHTO_
0.75	0.0011	0.0033	0.0031	0.94
1.00	0.0011	0.0050	0.0051	1.02
1.25	0.0011	0.0072	0.0083	1.15
1.50	0.0011	0.0099	0.0126	1.27
1.75	0.0011	0.0130	0.0180	1.39
2.00	0.0011	0.0167	0.0262	1.57

Table 4 indicates the ratio of the deep beam ultimate shear strength using ɛ_1-test_ and ɛ_1-AASHTO_ to the ultimate shear strength from the experiment. Using ɛ_1-test_ in place of ɛ_1-AASHTO_ obtained more accurate results for the ultimate shear strength of deep beams considering the average value in Table 4. Therefore, calibrating the value of ɛ_1-AASHTO_ with the experimental results is needed to enhance the results of ASHTO LRFD STM for RC deep beams.

**Table 4:** Comparison of ultimate shear strength of RC deep beams using ɛ_1-test_ and ɛ_1-AASHTO_ with test results.

No	a/d	P_AASHTO/_P_test_	P_ɛ1-test_/P_test_
1	0.75	1.09	0.95
2	1.00	1.02	1.03
3	1.25	1.14	1.04
4	1.50	1.12	0.94
5	1.75	1.35	0.94
6	2.00	1.31	0.95
**Average**		1.17	0.97

The a/d ratio mainly governed the behaviour of deep beams. Therefore, an empirical equation was proposed between the two ratios of and, ɛ_1-test_/ɛ_AASHTO_ as shown in Eq (8). ɛ_1-test_: principal tensile strain of strut from the test.

**Fig 12:**
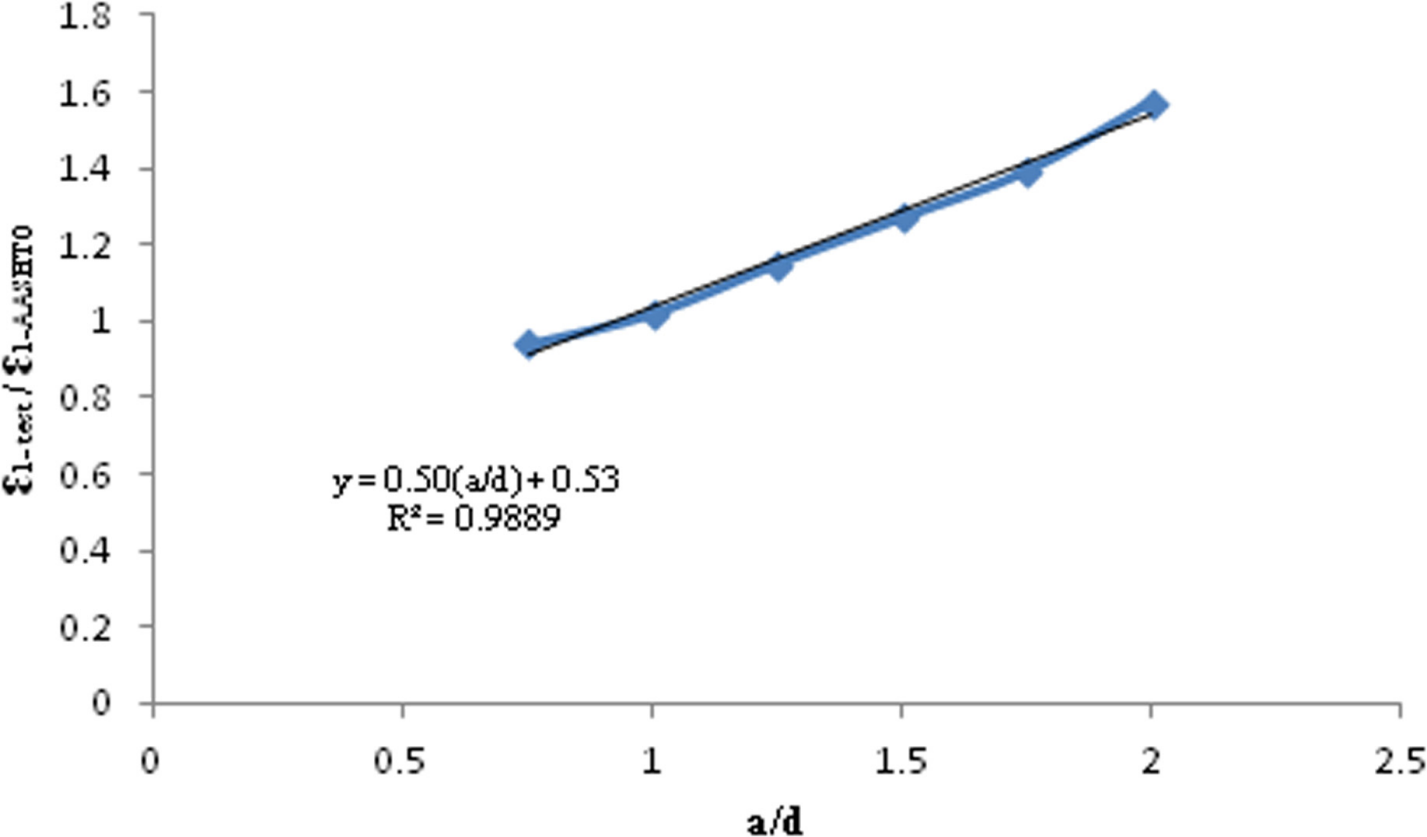
Empirical relationship between ɛ_1-AASHTO and_ ɛ_1-test_ for RC deep beams with 0.75≤a/d≤2.00.

The data from Table 3 were plotted in Fig 12 to obtain Eq (8). The empirical Eq (8) was proposed to modify the principal tensile strain of the RC strut that AASHTO LRFD recommended for RC deep beams with 0.75 ≤ a/d ≤ 2.00.

Eqs (8) and (9) obtain ɛ_1-recommended_ based on ɛ_1-AASHTO_ and a/d. This empirical equation is confined to STM used in analyzing RC deep beams.

$$\text{R}=0.50(\frac{\text{a}}{\text{d}})+0.53$$ (8)

$$\varepsilon_{1-\text{recommended}}\;=\text{R}\times \varepsilon_{1-\text{AASHTO}}$$ (9)

Where,

R: modification ratio, ɛ_1-recommended_: principal tensile strain of strut recommended in this research, ɛ_1-AASHTO_: principal tensile strain of strut from AASHTO LRFD

## Conclusion

This study aimed to modify STM for RC deep beams through the modification of the strut effectiveness factor. Six RC deep beams with different a/d ratios were tested until failure. Based on the experimental results, the following conclusions are drawn:

1. The results of STM method recommended by AASHTTO LRFD (2012) in the range of 0.75≤a/d≤2.00 were averagely in better agreement with the experimental results than the results of ACI 318–11.
2. An empirical equation was proposed to modify the theoretical value of principal tensile strain value of strut from AASHTO LRFD for RC deep beams with 0.75 ≤ a/d ≤ 2.00. The strut effectiveness factor equation was then modified using this empirical equation.
3. The elastic behavior was dominant for the RC deep beams for applied load approximately up to 30% of the ultimate load.
4. The first flexural cracks appeared at the applied load that was approximately greater than 50% of the ultimate load.
5. For an equivalent applied load, the maximum width of the diagonal crack increased with the increase of the a/d ratio.

The empirical equation proposed for the principal tensile strain of strut is confined to STM used in analysis of RC deep beams. The proposed empirical equation in this research can be further explored through the consideration of the RC deep beams with a/d ratio below 0.75. The results of NLFEM showed lower accuracy than those of obtained by STM method from ACI 318–11 and AASHTO LRFD. However, the results of NLFEM may become more accurate with improved assumptions. The adoption of smeared crack model to attain crack morphology may improve the NLFEM results. The effect of size of deep beams on the proposed model could be further explored with conducting more extensive experimental program.
